# A new case of spastic paraplegia type 64 due to a missense mutation in the *ENTPD1* gene

**DOI:** 10.1038/s41439-018-0036-4

**Published:** 2019-01-11

**Authors:** Jean Mamelona, Nicolas Crapoulet, Alier Marrero

**Affiliations:** 1 Department of Neurology, Dr.-Georges-L.-Dumont University Hospital Center, 330, University Avenue Moncton, Moncton, NB E1C 2Z3 Canada; 2 Centre de Formation Médicale du Nouveau-Brunswick, 100 Des Aboiteaux Street, Moncton, NB E1A 7R1 Canada; 3 Molecular Genetics, Dr.-Alfred-Bastarache Laboratory, 37 Providence Street, Moncton, NB E1C 8X3 Canada

**Keywords:** Neuromuscular disease, Next-generation sequencing, Genetics research

## Abstract

Spastic paraplegia type 64 (SPG64; OMIM 615683) is a complicated form of hereditary spastic paraplegia (HSP) recently identified in individuals diagnosed with suspected neurodegenerative disease. Affected patients carry homozygous mutations in the ectonucleoside triphosphate diphosphohydrolase 1 gene (*ENTPD1*). Although they share common characteristics, affected individuals show slight discrepancies in some clinical aspects. At present, only two different cases of SPG64 have been diagnosed. More findings of genetic variation would be helpful to better understand the effect of mutations in the *ENTPD1* gene on the neurological condition of affected individuals. In this study, we examined a family with an individual diagnosed with suspected HSP based on clinical findings. DNA samples from the proband, her affected sister, and both parents were analyzed using next-generation sequencing. We used an in-house automated pipeline to detect potential neuromuscular disease-causing variants. Variants were confirmed by Sanger sequencing. After cosegregation analysis, the variant NM_001776.5:c.401T>G (p.M134R) of the *ENTPD1* gene was identified as a novel missense mutation linked to the phenotype of SPG64 in the proband and her sister, who showed similar and distinct clinical features compared with the two cases previously described in the literature.

## Spastic paraplegia: Novel mutation in new cases of rare disorder

Canadian researchers have found a novel mutation in new cases of hereditary spastic paraplegia (HSP), a group of rare neurodegenerative disorders which involve progressive lower limb spasticity. Alier Marrero of the Dr. Georges-L.-Dumont University Hospital Center, Moncton and colleagues examined a suspected HSP patient and her family. The patient had a speech disorder and abnormal gait from an early age and later became unable to walk. Her sister had milder forms of the same symptoms. Marrero’s team discovered a new mutation in the *ENTD1* gene in both sisters and found that their parents were unaffected carriers. *ENTD1* mutations have been linked with spastic paraplegia type 64 (SPG64), a form of HSP that has been diagnosed in only two other clinical cases. Further research will clarify how these mutations cause the symptoms associated with SPG64.

## Introduction

Hereditary spastic paraplegias (HSPs) are a group of rare and heterogeneous genetic neurodegenerative diseases. They are characterized by length-dependent distal axonal degeneration, which causes a loss of function of the corticospinal tract, resulting in progressive lower limb spasticity. Clinically, HSPs are classified into two broad categories, uncomplicated and complicated, based on the manifestation of additional features, such as ataxia, delayed psychomotor development, dysarthria, mental retardation, and visual defects^1^. For complicated HSPs, the clinical symptoms might resemble between affected patients. Therefore, it is very difficult to make a reliable diagnosis of a specific HSP based solely on individual history, physical and physiological examinations, and magnetic resonance imaging (MRI). Like many other hereditary neurodegenerative disorders, the final diagnosis of HSP is usually made after genetic testing confirms the presence of a mutation known to be linked to the suspected specific type of the disease.

By using whole-exome sequencing in combination with network analysis, researchers recently identified in individuals diagnosed with suspected neurodegenerative disease a new form of complicated HSP called autosomal recessive spastic paraplegia type 64 (SPG64; OMIM 615683)^2^. Affected patients carry homozygous mutations in the ectonucleoside triphosphate diphosphohydrolase 1 gene (*ENTPD1*). Clinically, they share common characteristics such as abnormal gait, lower limb weakness, and intellectual disability. However, they show slight discrepancies in some clinical aspects, notably onset and further symptoms. At present, only two different clinical cases of SPG64 have been diagnosed^2^. Like for many other HSPs, there is a need for more findings for SPG64, specifically genetic variation^3^, to better understand mutations in the *ENTPD1* gene as well as their effects on the neurological condition of the affected individuals.

In this study, we examined a family with an individual diagnosed with suspected HSP based on individual history and clinical findings. DNA samples from the proband, her affected sister, and both parents were analyzed using next-generation sequencing (NGS) and an in-house automated pipeline to detect potential neuromuscular disease-causing variants. This study reports a novel mutation in the *ENTPD1* gene. Bioinformatics analyses indicated a damaging impact of this mutation on the function of the ENTPD1 protein. The novel mutation is linked to SPG64, with affected individuals showing similar and distinct clinical features compared to findings described previously in the literature.

## Materials and methods

### Participants

**Fig. 1:**
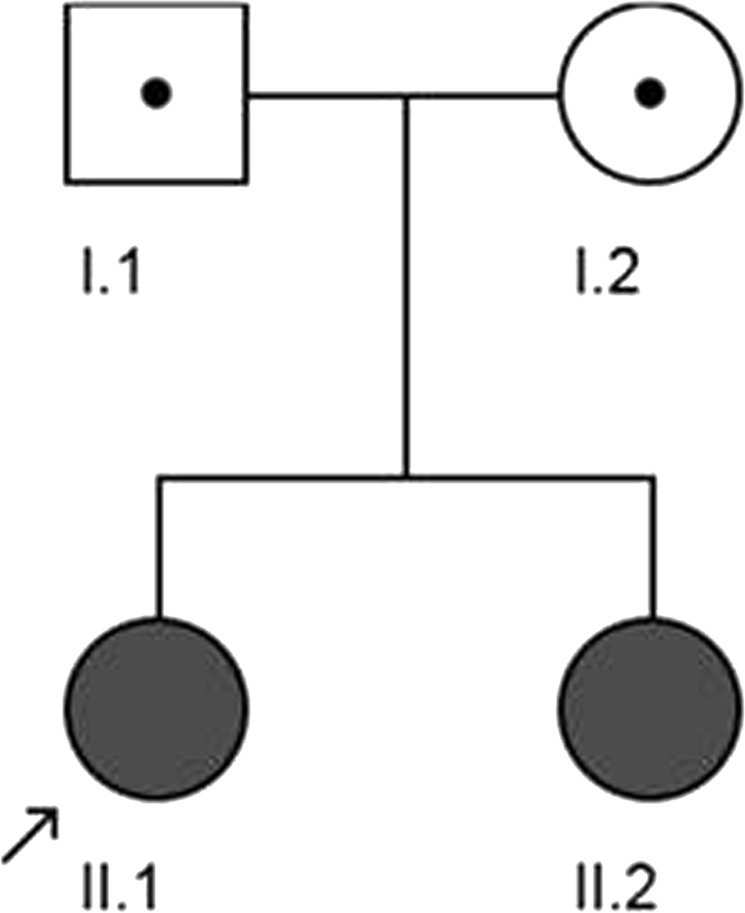
Pedigree of participants in the study of a family with an individual diagnosed with suspected hereditary spastic paraplegia (HSP). Solid symbol indicates affected individuals; doted open symbol indicates unaffected carriers. Arrow indicates the proband

This study complied with the Declaration of Helsinki Principles and was approved by the Research Ethic Board of the Vitalité Health Network in the province of New Brunswick, Canada. The participants were informed and asked to give their consent by a signed written form before beginning the study. The proband has been seen by physicians since the age of 14 months for various neurological issues related to her unstable gait. She was diagnosed with suspected HSP at the age of 17 years. Her parents were informed about the need for genetic testing to make a reliable diagnosis. Thereafter, the rest of her family, including her sister and both parents, were asked to participate in genetic testing (Fig. 1). All participants received comprehensive physical examinations. Their blood samples were collected for further whole-exome sequencing. The proband and her sister additionally received MRI examinations.

### Exome library preparation and sequencing

The genomic DNA of each participant was extracted from whole blood using a MagNA Pure Compact system and MagNA Pure Compact Nucleic Acid Isolation Kit I (Roche Life Science, Indianapolis, IN, USA) according to the manufacturer’s instructions. DNA was quantified using a TaqMan RNase-P Quantitation Kit (Thermo Fisher Scientific, Waltham, MA, USA) on an LC480 LightCycler system (Roche Life Science). Genomic DNA (100 ng) was subsequently subjected to whole-exome DNA library construction using the Ion AmpliSeq Exome panel (Thermo Fisher Scientific) essentially as described in the manufacturer’s protocol, with barcode incorporation. The libraries were then quantified by using an Ion Library TaqMan Quantitation Kit (Thermo Fisher Scientific) on an LC480 LightCycler system (Roche Life Science). The concentration of the exome libraries was diluted to 8 pM, and the libraries were pooled to analyze two samples on an Ion HI-Q PI Chip v3. Template preparations consisting of emulsion PCR, enrichment of beads containing template, and chip loading were performed by using the Ion PI HI-Q Template OT2 Kit and Ion One Touch 2 system (Thermo Fisher Scientific) according to the manufacturer’s instructions. Samples were loaded on an Ion HI-Q PI Chip v3 and placed onto the Ion Proton instrument (Thermo Fisher Scientific) together with an Ion PI HI-Q sequencing 200 Kit (Thermo Fisher Scientific) and sequenced for 520 cycles according to the manual.

### Bioinformatics analysis, Sanger sequencing, and cosegregation

After Ion Proton sequencing, low-quality reads and adaptor sequences were filtered out using the Torrent suite software v.4.4.2 (Thermo Fisher Scientific). Subsequently, Ion Reporter server v.4.4 (Thermo Fisher Scientific) was used to align the remaining high-quality sequencing reads to the reference human genome (hg19) and to annotate the identified single-nucleotide polymorphisms and InDels. Variant interpretation for trio analysis was then realized with the Genomatix GeneGrid software (Genomatix, Ann Arbor, MI, USA) according to the patient phenotype. IGV software was used to view the short-read alignments and validate the candidate single-nucleotide polymorphisms and InDels. Nonsynonymous variants were then evaluated using four algorithms, PhyloP, Grantham, SIFT, and PolyPhen, to predict the pathogenic potential of the variants. All suspected neuromuscular disease-causing mutations found by NGS were validated by direct Sanger sequencing. DNA sequences were obtained from the University of California Santa Cruz (UCSC) Genome Browser. Predesigned primers were directly purchased from Thermo Fisher Scientific according to accession number HS00321918 (ENTPD1_forward primer: TCCAGTGAATTACCCTCTAAGTACAAT; ENTPD1_reverse primer: CCCATAGGGAGATGCACAAGG). Amplicons were sent to an external laboratory for Sanger sequencing. All family members were subsequently sequenced to perform cosegregation analysis.

## Results

### Onset

The proband started to walk at the age of 14 months. She showed an ataxic gait, walked on tip toe, was very active, and frequently fell down. Her sister started to walk normally at the age of 10 months. No significant neurological issue was detected for her sister until the age of 3 years, when she started to show an ataxic gait.

### Further symptoms

The proband began to show dysarthria from the moment of her development where she was supposed to speak in understandable language, around the age of 3 years. She first walked with support at the age of 9 years and showed a bilateral Tendelenburg gait at the age of 14 years. She became non-ambulatory at the age of 15 years. Her sister never showed speech issues until the age of 11 years, when she began talking with a nasal voice. She then showed dysarthria at the age of 12 years and began to show a wide base gait at the age of 15 years (Table 1). The last time she was seen by physicians (21 years), she was still walking independently. Both sisters showed spasticity in the lower limbs, absence of deep tendon reflex, Babinski reflex, gait ataxia, aggressiveness, and moderate learning disability. No muscular atrophy or fasciculation was observed. Nerve conduction studies and electromyographic (EMG) tests showed normal results for the proband. However, decreased right ulnar compound muscle action potential with mildly increased insertional activity and decreased recruitment on needle EMG of an ulnar-innervated muscle were observed for her sister, suggesting that she is in the early stage of developing a polyneuropathy. Sensory examination showed that both sisters exhibit low vibration sensing and proprioception.

### Brain imaging

Cerebral MRI showed no significant changes in the brain for both the proband and her sister.

### Confirmation of mutation of the *ENTPD1* gene by genetic testing

**Fig. 2:**
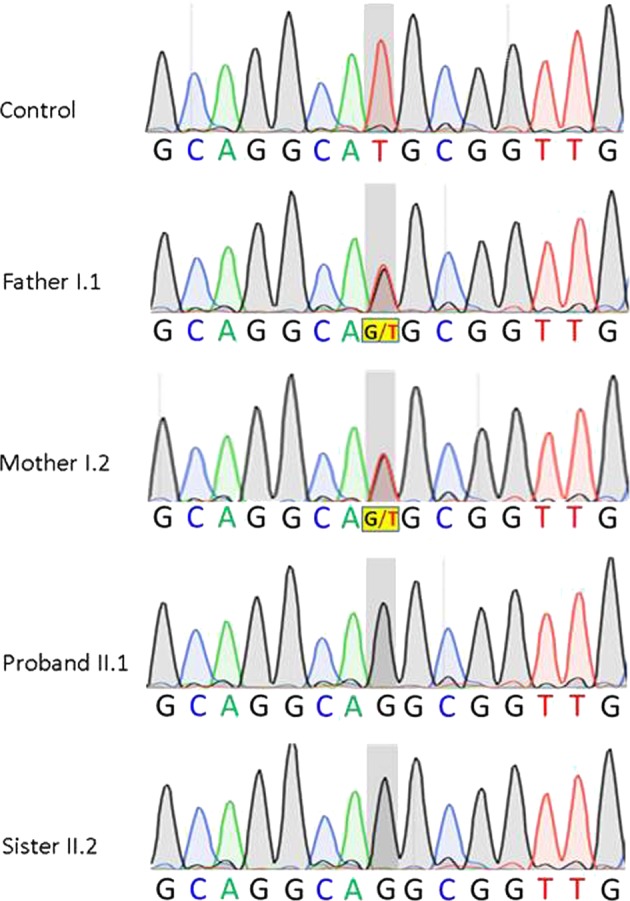
Electropherograms showing the co-segregation of the homozygous mutation c.401T>G in the *ENTPD1* gene to the phenotype observed in proband and her sister. Proband II.1 and her sister II.2 are carrying homozygous alleles (G/G) linked to the phenotype whereas non-affected parents (father I.1 and mother I.2) are carrying heterozygous alleles (G/T). Location of mutation highlighted in grey

**Fig. 3:**
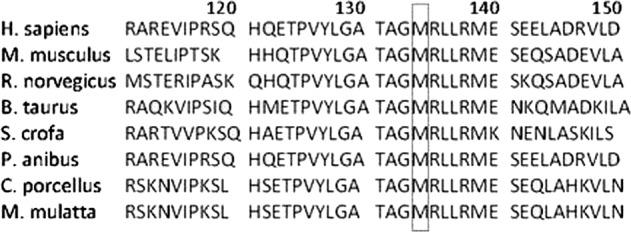
Partial results from conservation analysis showing that the residue 134 in the ENTPDase-1 is highly conserved among different species of vertebrates

Using the stepwise variant-filtering strategy described above, three candidate mutations were identified in all participants (Table 2). Sanger sequencing confirmed the presence of the novel variant NM_001776.5:c.401T>G (p.M134R) in the *ENTPD1* gene, found in both affected sisters as homozygous, while both parents were heterozygous (Fig. 2). The two other variants, NM_000493.3:c.256G>A (p.G86R) in the *COL10A1* gene and NM_024301:c.427C>A (p.R143S) in the *FKRP* gene (See Table 2), did not cosegregate with the phenotype of suspected HSP in this family, as they were similarly detected in both affected daughters and their unaffected parents. Bioinformatics analyses using the aforementioned algorithms showed that nucleotide T at position 401 of the *ENTPD1* gene is highly conserved across 44 vertebrate species (PhyloP at 2.21; See Fig. 3). Analysis of the amino acid substitution from methionine (M) to arginine (R) at position 134 of the ENTPD1 protein suggested that the mutation will have a significant impact on the structure of the ENTPD1 protein (Grantham at 91). The prediction also indicated a damageable impact of this mutation on the function of the ENTPD1 protein (SIFT at 0 and PolyPhen2 at 1). The results of the analysis obtained from VarSome (https://varsome.com/variant/hg19/NM_001776.5%3Ac.401T%3EG) showed a verdict of uncertain significance, but computational results suggesting a pathogenic consequence were obtained from eight various prediction software packages, including DANN, GERP, dbNSFP.FATHMM, MetaLR, MetaSVM, MutationAssessor, MutationTaster, and PROVEAN (vs no benign predictions). In silico analyses of the potential impact of this variant on RNA splicing using MutPred Splice v1.3.2 gave a result of splice neutral variant. However, analysis using Human Splicing Finder V3.1 predicted a creation of an exonic splicing silencers site and thus a potential alteration of splicing.

**Table 1:** Main clinical features for proband and her sister

	Proband	Sister
Onset, age	Ataxic gait, 14 months	Ataxic gait, 3 years
	Tip toe walking, 14 months	
	Frequent falls, 14 months	
Further symptoms, age	Dysarthria, 3 years	Dysarthria, 11 years; nasal voice, 12 years
	Walking with support, 9 years Non-ambulatory, 15 years	Walking independently
	Bilateral Tendelenburg gait, 14 years	Wide base gait, 15 years

**Table 2:** Candidate exome sequence variants filtered by database

Chr. position	Gene	Transcript ID	Mutation	Mode, inheritance	Mutation type	dbSNP ID	ClinVar accession #	SIFT	Polyphen2	Grantham	PhyloP
Chr10:97602239	*ENTPD1*	NM_0017776.5	c.401T>G, p.M134R	Homozygous, AR	NS, SNV	rs1480686371	Novel	0	1	91.0	2.21
Chr6:116443023	*COL10A1*	NM_000493.3	c.256G>A, p.G86R	Homozygous, AD	NS, SNV	rs145214720	SCV000459720.2	ND	1	125.0	2.77
Chr19:47259134	*FKRP*	NM_024301.4	c.427C>A, p.R143S	Heterozygous, AR	NS, SNV	rs148206382	SCV000797516.1	ND	ND	110.0	1.16

Chr. positions are established on Assembly GRCh37

*Chr* chromosome, *AR* autosomal recessive, *AD* autosomal dominant, *NS* non-synonymous, *SNV* single-nucleotide variants, *ND* not determined

## Discussion

HSPs are a group of heterogeneous genetic neurodegenerative diseases. Since the first report of HSP-causing mutations 20 years ago, new mutations for this group of diseases have continued to be reported^1,3^. Researchers recently reported mutations in the *ENTPD1* gene linked to a new complicated form of HSP called autosomal-recessive spastic paraplegia type 64^2^. The *ENTPD1* gene encodes the multipass membrane protein ectonucleoside triphosphate diphosphohydrolase 1 (ENTPDase-1). The latter catalyzes dephosphorylation reactions of extracellular nucleotides involved in the regulation of purinergic neurotransmission in the nervous system^4,5^. The protein comprises three topological domains: two small cytoplasmic domains at the N- and C-termini and a large extracellular loop domain that contains the catalytic site. This extracellular domain is composed of 441 amino acids spanning from positions 38–478 [UniProtKB—P49961 (ENTP1_HUMAN); https://www.UniProt.org/UniProt/P49961]. Both mutations in the *ENTPD1* gene reported at present involve protein secondary structure changes in this domain, namely, p.E181X and p.G217R^1^. This highlights the significance of a single amino acid change in this extracellular domain in the alteration of the normal function of ENTPDase-1.

A novel variant c.401T>G was recently reported in the dbSNP database with the assigned number rs1480686371, but no information on a related condition was given. Our team first submitted this variant to the ClinVar database along with the assertion of the related condition under accession number SCV000804555. Accordingly, in this article we consider this mutation a novel phenotypic variant. In terms of amino acid structure, this variant leads to a single change, p.M134R, in the extracellular domain of ENTPDase-1 [UniProtKB—P49961 (ENTP1_HUMAN); https://www.UniProt.org/UniProt/P49961]. This change is located at the third amino acid of the fifth α-helix in chain A of the crystal structure of ENTPDase-1. This helix spans from position 132 to 140 with the following sequence: AGMRLLRME (PDBsum Entry 3zx3; http://www.ebi.ac.uk/thornton-srv/databases/cgi-bin/pdbsum/GetPage.pl)^6^. As recently demonstrated by molecular dynamics simulations, the dissociation energy of the helical structure is related to amino acid preference. Accordingly, this change in the amino acid sequence might impair the electrostatic interactions between amino acid residues and destabilize the helical structure because the positively charged amino acid arginine is present at successive positions, 133 and 134, in the resulting mutant structure^7^. These structural implications are consistent with our bioinformatics analysis, which showed that this novel variant is located at a highly conserved position among several species and suggested significant damage to protein structure and function.

The enzyme ENTPDase-1 is part of a group of nucleotide triphosphate diphosphohydrolases that are involved in a purinergic cascade that, in turn, regulates all aspects of purinergic signaling. The latter is involved in almost all neurotransmission functions in body and depends on the level of neurotransmitters available within the extracellular area, notably ATP and adenosine. More specifically, ENTPDase1 is involved in cell-surface catabolism of ATP and ADP with AMP as the final product^4,5^. AMP can then be broken down by other enzymes of the purinergic cascade to adenosine and inosine. As mentioned above, the deleterious effect of the novel variant c.401T>G may affect the normal function of ENTPDase-1, potentially leading to an imbalance of extracellular levels of the neurotransmitters ATP and adenosine and consequently disturbing purinergic neurotransmission, which is thought to be responsible for the etiology of SPG64.

On the basis of the early symptoms of the proband and her sister, they were diagnosed with suspected neurological diseases. The results of further physical examinations narrowed the investigation to a diagnosis of suspected HSP. After NGS sequencing and cosegregation analysis, the variant c.401T>G (p.M134R) in the *ENTPD1* gene, for which both sisters were homozygous, was identified as a mutation responsible for a complicated form of HSP, autosomal-recessive spastic paraplegia type 64 (SPG64). This is the third case of mutations in the *ENTPD1* gene linked to SPG64 to be reported in the literature. Based on the three mutations reported so far, SPG64 is linked to potential protein function impairment caused by an amino acid sequence change in the extracellular loop domain that contains the catalytic site^4,5^. Further studies are needed to elucidate the mechanisms and details of the effects of those mutations in altering purinergic neurotransmission and the neurological conditions of patients.

Current knowledge has shown that early symptoms detected at onset as well as further clinical features and their severity may differ among individuals affected by this disease, even within the same family (Supplementary Table S1). For all three cases of SPG64, final diagnoses have been made on the basis of results of DNA testing. This fact highlights the significance of genetic screening using very useful NGS technologies for the diagnosis of HSP and other similar genetic neurodegenerative diseases. SPG64 can be very invasive and greatly affect quality of life. To cautiously manage the risk of this disease, there is a need for DNA testing of family members to assist genetic counseling for better reproductive decision-making.

## Supplementary information

Table S1
