# Supplementary material for: A new case of spastic paraplegia type 64 due to a missense mutation in the ENTPD1 gene
Source: Hum Genome Var. 2019 Jan 11;6:5. doi: 10.1038/s41439-018-0036-4 (PMC6329766; doi:10.1038/s41439-018-0036-4)
Supplement: Supplementary file 1 — Table S1 [file 41439_2018_36_MOESM1_ESM.pdf]

Table S1. Phenotype of all patients diagnosed with SPG64 at present

Subjects	Family 1 - IV.2	Family 1 - IV.4	Family 2 - VI.1	Family 2 - VI.6	Family 3 - II.1	Family 3 - II.2
Age at onset	3.5 yrs	4 yrs	1 yrs	1 yrs	1 yrs	3 yrs
Age at last examination	15 yrs	10 yrs	22 yrs	11 yrs	17 yrs	19 yrs
Sex	M	M	M	F	F	F
Signs at onset	Abnormal gait	Abnormal gait	Unsteady gait	Unsteady gait	Abnormal gait	Abnormal gait
Spasticity	+	+	-	-	+	+
Dysarthria	-	-	+	+	+	+
Disability stage Motor deficit (at last examination)	Nonambulatory	Can walk, with support	Can walk, with support	Can walk, unsupported, with abnormal gait	Nonambulatory	Can walk, unsupported, with abnormal gait
Cerebellar signs	NR	NR	+	+	+	+
Ophthalmologic signs	NR	NR	Congenital cataract	-	-	-
Reflexes (lower limbs)	Increased deep tendon reflex	Increased deep tendon reflex	Absent reflexes	Normal	Absent reflexes	Absent reflexes
Amyotrophy	+	-	+	+	-	-
Skeletal deformities	Pes equinovarus	-	-	-	-	-
Brain MRI	NR	NR	Mild white matter changes	Mild white matter changes	Normal	Normal
Intellectual disability	Borderline intelligence	Borderline intelligence	Moderate intellectual disability	Moderate intellectual disability	Moderate intellectual disability	Moderate intellectual disability
Other signs	Aggressiveness, delayed puberty, microcephalic	Aggressiveness, delayed puberty, microcephalic	NR	NR	Aggressiveness	Aggressiveness, nasal voice

In order to facilitate the tracking for families for future studies on SPG64-causing mutations, families have assigned with new numbers. However, hierarchic numbers in the original pedigrees have been kept to facilitate further verification. Family 1: Family 1242 from Novarino et al., 2014; Family 2: Family 1800 from Novarino et al., 2014; Family 3: The family from the present study. NR, Not reported
